# The Inducible *lac* Operator-Repressor System Is Functional in Zebrafish Cells

**DOI:** 10.3389/fgene.2021.683394

**Published:** 2021-06-18

**Authors:** Sierra S. Nishizaki, Torrin L. McDonald, Gregory A. Farnum, Monica J. Holmes, Melissa L. Drexel, Jessica A. Switzenberg, Alan P. Boyle

**Affiliations:** ^1^Department of Human Genetics, University of Michigan, Ann Arbor, MI, United States; ^2^Department of Computational Medicine and Bioinformatics, University of Michigan, Ann Arbor, MI, United States

**Keywords:** zebrafish, *lac* operator-repressor system, luciferase, GFP, reporter

## Abstract

**Background:**

Zebrafish are a foundational model organism for studying the spatio-temporal activity of genes and their regulatory sequences. A variety of approaches are currently available for editing genes and modifying gene expression in zebrafish, including RNAi, Cre/lox, and CRISPR-Cas9. However, the *lac* operator-repressor system, an *E. coli lac* operon component which has been adapted for use in many other species and is a valuable, flexible tool for inducible modulation of gene expression studies, has not been previously tested in zebrafish.

**Results:**

Here we demonstrate that the *lac* operator-repressor system robustly decreases expression of firefly luciferase in cultured zebrafish fibroblast cells. Our work establishes the *lac* operator-repressor system as a promising tool for the manipulation of gene expression in whole zebrafish.

**Conclusion:**

Our results lay the groundwork for the development of *lac-*based reporter assays in zebrafish, and adds to the tools available for investigating dynamic gene expression in embryogenesis. We believe this work will catalyze the development of new reporter assay systems to investigate uncharacterized regulatory elements and their cell-type specific activities.

## Background

Experimental approaches for the study of transcriptional regulation by cis-regulatory elements *in vivo* require methods for both genetically modifying cells or organisms, and for measuring expression levels of specific genes. Zebrafish (*Danio rerio*) is an ideal model organism for investigating the spatio-temporal-specific regulation of gene expression throughout the developing embryo as it satisfies the requirements for ease of genetic manipulation and expression readout. Microinjection of DNA into fertilized embryos allows for the simple and effective delivery of genome-modification tools, such as *Tol2* transposons, that mediate genomic integration of constructed expression cassettes. Additionally, the transparency of zebrafish embryos facilitates the observation of fluorescent signals from reporter genes within live cells and tissue. Due to its benefits as a model organism, many technologies for studying gene function have been developed in zebrafish, including Cre/lox (Langenau et al., 2005), tamoxifen-inducible Cre (Hans et al., 2009), the Tet-On system (Campbell et al., 2012), RNAi (Dodd et al., 2004; Kelly and Hurlstone, 2011), and more recently, CRISPR based-methods (Hwang et al., 2013). However, the use of the *lac* operator-repressor system, a tool that functions transiently in a native context with minimal disruption of local regulation compared to many of the aforementioned methods, has yet to be demonstrated in zebrafish.

The *lac* operator-repressor system is an inducible repression system established from studies of the *lac* operon in *Escherichia coli* (*E. coli*) that regulates lactose transport and metabolism (Jacob and Monod, 1961). The Lac repressor (LacI) binds specifically to a *lac* operator sequence (*lacO*), inhibiting the *lac* promoter and *lac* operon expression through steric hindrance (Brown et al., 1987). Addition of the allosteric inhibitor Isopropyl β-d-1-thiogalactopyranoside (IPTG) to cells frees the *lac* operon to express its associated gene by inhibiting the binding of LacI to *lacO* sequences. The use of IPTG with the *lac* operator-repressor allows for inducible reversal of transcriptional repression.

Since its discovery in prokaryotes, the *lac* operator-repressor system has been modified for use in eukaryotic organisms to study the regulation of gene transcription (Brown et al., 1987; Hu and Davidson, 1987; Liu et al., 1989; Bowler et al., 1992). Experiments in mammalian cell lines from mouse, monkey, and human (Brown et al., 1987; Hu and Davidson, 1987; Figge et al., 1988; Liu et al., 1989), as well as in whole mouse (Wyborski et al., 1996), demonstrate the utility of the *lac* operator-repressor system. It has also successfully been applied in cell lines and whole Xenopus and axolotl animals, suggesting that this system can be utilized in a wide range of organisms (Hu and Davidson, 1988; Whited et al., 2012). Modifications to the *lac* operator-repressor system has allowed for constitutive, ubiquitous expression (Brown et al., 1987; Biard et al., 1992; Myung et al., 2020), visually assessed output (Figge et al., 1988; Liu et al., 1989), and the ability to study both gene repression and activation (Lee et al., 2017; Vander Schaaf et al., 2019), emphasizing its flexibility for studying gene expression dynamics. The ability of IPTG to relieve repression in the *lac* system makes it a more adaptable tool for studying the temporal dynamics of gene expression, compared to constitutively active or repressed reporter gene systems.

In this paper, we provide evidence that the *lac* operator-repressor system can function in the zebrafish fibroblast cell line PAC2, adding a versatile new tool for the study of zebrafish genetics and transcriptional regulation. The results in a zebrafish cell line support the potential functionality of the *lac* operator-repressor system to function in whole zebrafish.

## Results

### The CMV Enhancer Shows Widespread Expression in Zebrafish

**FIGURE 1:**
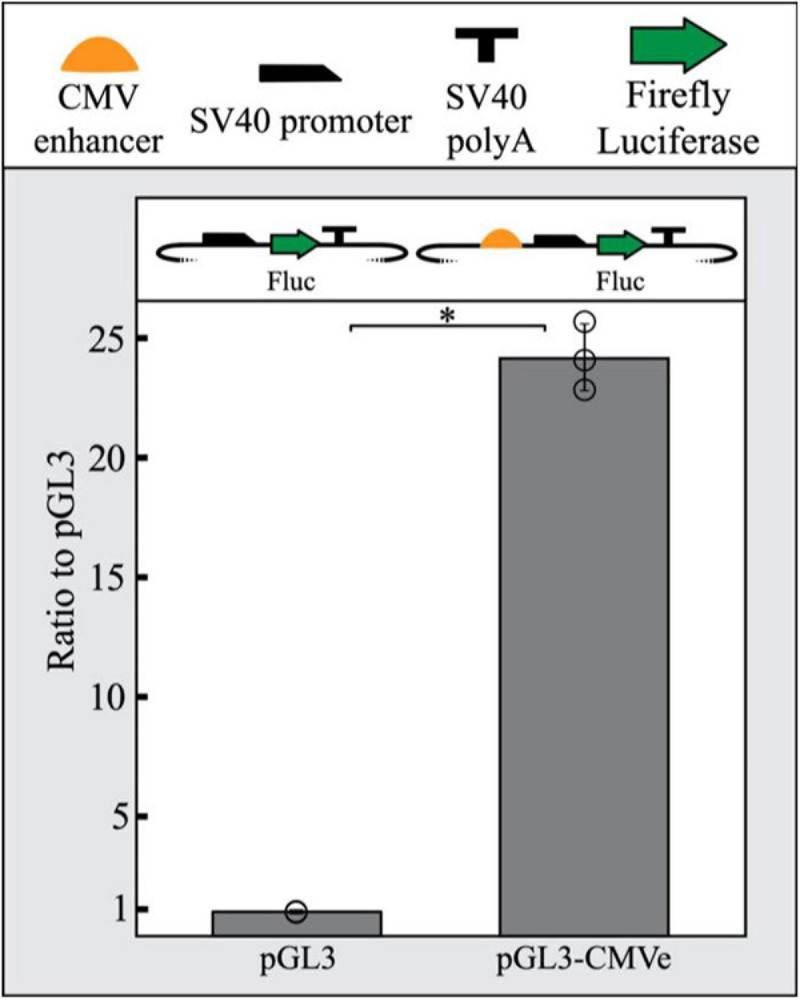
The CMV enhancer drives strong reporter gene expression in PAC2 cells. The CMV enhancer shows a 24-fold increase in luciferase activity in PAC2 zebrafish fibroblast cells compared to a SV40 promoter-only plasmid. Error bars represent standard deviation of 3 biological replicates. Statistical significance was determined using a Student’s two tailed *t*-test ^∗^*P* < 0.01. Points represent values for all 3 replicates in each condition. All plasmid components are detailed as symbols above the figure. The TSS starts where the SV40 promoter begins to slope downward.

To promote the repression of a reporter gene in our assay, we sought to increase LacI expression in transfected cells by including a strong enhancer driving LacI. The CMV enhancer is frequently used in reporter vector construction across a wide range of studies due to its robust and constitutive promotion of gene expression. This includes zebrafish where the CMV enhancer has been previously shown to yield strong widespread expression (Su et al., 2008; Jia et al., 2020). We demonstrated that the CMV enhancer functions in PAC2 cells by inserting a CMV enhancer and SV40 minimal promoter upstream of luciferase in a pGL3 plasmid. Relative luciferase output of the CMV-enhanced SV40 pGL3 plasmid was compared to a pGL3 plasmid containing only a minimal SV40 promoter. The CMV enhancer was able to drive a 24-fold increase in luciferase expression compared to the promoter-only control, suggesting the CMV enhancer is also able to function as a strong enhancer in PAC2 cells specifically (Figure 1).

Validation of the strong enhancer activity of CMV in whole zebrafish was completed using an enhancer assay expressing eGFP fluorescence injected into zebrafish embryos (Kawakami, 2007). Two constructs were evaluated; one containing a CMV enhancer upstream of an eGFP reporter gene, and one with only a minimal promoter. While no detectable level of eGFP activity in the promotor-only control was observed (0/76 GFP + at 24 h) (Supplementary Figure 1A), composite brightfield and GFP images of 24 and 48 h post-fertilization embryos injected with the CMV enhancer assay showed strong widespread eGFP expression (63/69 GFP^+^ at 24 h, 49/49 GFP^+^ at 48 h) (Supplementary Figure 1B). Together these data provide solid evidence that CMV enhancer-driven LacI expression in whole zebrafish is feasible.

### The *lac* Operator-Repressor System Is Functional in the PAC2 Zebrafish Cell Line

**FIGURE 2:**
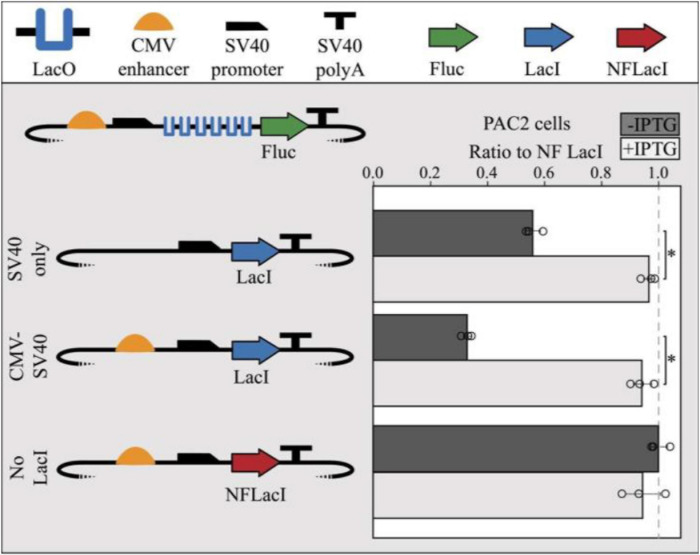
Co-transfection of LacI-expressing modules with repressible reporter modules result in LacI-mediated repression in PAC2 cells. SV40 promoter-only driven expression of LacI shows moderate repression (40%) and CMV enhancer-driven expression of LacI shows high repression (70%) of a repressible module containing 6x LacO sites. Expression of non-functional LacI (NFLacI, frameshift mutant) shows no repression and all modules showed maximal reporter expression in the presence of 1 mm IPTG. Error bars represent standard deviation of replicates (*n* = 3). Points represent values for all 3 replicates in each condition. The dashed line shows the NF LacI IPTG- negative control. The TSS starts where the SV40 promoter begins to slope downward. Statistical significance was determined using a Student’s two tailed *t*-test ^∗^*P* < 0.001.

To test the functionality of the *lac* operator-repressor system in zebrafish, a repressible reporter plasmid containing 6 *lac* operators in the 5′UTR of the firefly luciferase gene and a LacI-expressing plasmid were co-transfected into PAC2 cells. When a plasmid expressing a non-functional LacI (NFLacI) gene was co-transfected, no repression was observed (Figure 2), whereas a plasmid expressing CMV enhancer-driven levels of LacI resulted in about 65% repression. An intermediate level of repression (∼40%) was observed when LacI was expressed from a plasmid containing only a SV40 minimal promoter, indicating that the extent of repression correlates with LacI levels in the cell. Addition of IPTG to the cells resulted in full relief of repression in all cases. Interestingly, increasing concentrations of IPTG inversely correlates with lac expression, potentially due to an adverse effect of IPTG on cell viability, or cell recovery and expression following transfection (Supplementary Figure 2). However, the IPTG dosing curve was only assessed in K562, and the experiment would need to be repeated to determine a toxicity profile in zebrafish cells. We further demonstrated that repression is linked to LacI levels when co-transfecting half the concentration of LacI into human K562 cells which contributed to reduced levels of luciferase repression (∼67%), approximately 30% less repression than cells transfected with equal LacI concentrations (∼51%) (Supplementary Figure 2). This indicates that LacI is responsible for repression of luciferase expression in these cells.

To validate that PAC2 repression levels are comparable to previously published data (Deans et al., 2007) and in a human cell line, the LacI experiment was replicated in the K562 human cell line. The performance of the *lac* operator-repressor system was nearly identical in PAC2 and K562 cells when both cell types were co-transfected with the same plasmid mixture (Supplementary Figure 3). The PAC2 and K562 cells showed around 60–65% repression when co-transfected with a molar equivalent of the CMV enhancer-driven LacI containing plasmid (∼400 ng), and roughly 10–20% repression with a similar molar equivalent of SV40 promoter-only driven LacI-expressing plasmid. These results demonstrate that the *lac* operator-repressor system functions in PAC2 cells at a comparable level to human K562 cells, and suggests this system may function broadly within different cell lines.

## Discussion

Zebrafish are a commonly used model organism for studying the spatio-temporal dynamics of *cis-*regulatory element activity and gene function. However, the flexible and widely used *lac* operator-repressor system has previously been untested in zebrafish. Here we demonstrate that the *lac* operator-repressor system functions in zebrafish cells, consistent with observed activity in other model eukaryotic systems.

For the development of a reporter system in whole organisms like zebrafish, it is critical to demonstrate non-tissue specific activity of an enhancer to provide robust output for single-cell reporter signal detection. The CMV enhancer is routinely used in reporter assays to drive strong and widespread gene expression (Su et al., 2008; Jia et al., 2020). We provide quantitative evidence that the CMV enhancer robustly increases luciferase gene expression over a promoter-only control plasmid in zebrafish fibroblast cells.

Changes in expression of the repressor protein LacI are inversely related to changes in reporter expression. This response appears to provide a level of repression directly related to the LacI level, rather than functioning as an on/off switch. This will allow for a more nuanced measure of *lac* regulatory control. For studies requiring a tight amount of gene repression, such as those in which gene dosage is critical, repression can be further improved through an increased molar ratio of LacI repressor plasmid (Supplementary Figure 3; Hu and Davidson, 1987). Previous studies have also demonstrated that doubling the number of LacO sites can decrease repression by 50% (Lee et al., 2017). Upon addition of IPTG, luciferase signal was recovered to the level of a non-functional LacI control, indicating that robust repression is completely reversible at low IPTG concentrations (Supplementary Figure 4). This was equally the case in both human and zebrafish cells, suggesting that, at low concentrations, the potentially adverse effects of IPTG on the expression of the luciferase vectors is minimized, if not altogether mitigated. The pronounced response to IPTG treatment, as well as minimal toxicity in a zebrafish cell line, suggest the *lac* operator-repressor system is a viable tool for use in whole zebrafish.

*Lac* operator-repressor systems can be used to control endogenous gene expression without interrupting native regulatory processes, as *lacO* sites can be inserted in benign regions, such as introns and UTRs. Transcriptional inhibition of RNA polymerase by steric hindrance can achieve repression without introducing artificial modifications to the locus and causing prolonged alterations in regulatory behavior. This is in contrast to other systems that achieve transcriptional control by tethering a protein domain with activating or silencing effects through chromatin modification, or other endogenous mechanisms. Specificity of repression is also less of a concern compared to novel CRISPRi methods known for off-target effects (Gilbert et al., 2014). As demonstrated by the REMOTE-control system, the lac system can be utilized in conjunction with Tet-related systems to drive both activation and repression of a single loci, bringing additional flexibility to zebrafish studies (Lee et al., 2017). This system allows for time-controlled experiments, where a reporter gene is repressed during a limited time window, making it a crucial tool for replicating the restriction of gene expression during development.

## Methods

### Plasmid Design

CMV-SV40 enhancer-promoter luciferase plasmids were generated by restriction digestion to insert a CMV enhancer and a minimal SV40 promoter, or only a minimal SV40 promoter, upstream of a luciferase reporter molecule in the context of a pGL3 plasmid (Promega, E1751). The sequence of the CMV-SV40 promoter is as follows: GGCATTGATTATTGACTAGTTATTAATAGTAATCAATTA CGGGGTCATTAGTTCATAGCCCATATATGGAGTTCCGCG TTACATAACTTACGGTAAATGGCCCGCCTGGCTGACCGC CCAACGACCCCCGCCCATTGACGTCAATAATGACGTATG TTCCCATAGTAACGCCAATAGGGACTTTCCATTGACGTC AATGGGTGGAGTATTTACGGTAAACTGCCCACTTGGCA GTACATCAAGTGTATCATATGCCAAGTCCGCCCCCTATTG ACGTCAATGACGGTAAATGGCCCGCCTGGCATTATGCCC AGTACATGACCTTACGGGACTTTCCTACTTGGCAGTACAT CTACGTATTAGTCATCGCTATTACCATGGACTTGCATCTC AATTAGTCAGCAACCATAGTCCCGCCCCTAACTCCGCCC ATCCCGCCCCTAACTCCGCCCAGTTCCGCCCATTCTCCG CCCCATGGCTGACTAATTTTTTTTATTTATGCAGAGGCC GAGGCCGCCTCTGCCTCTGAGCTATTCCAGAAGTAGTGA GGAGGCTTTTTTGGAGGCCTAGGCTTTTGCAAAAAG CTC.

*Lac* operator-repressor system plasmids were created using the EMMA golden gate assembly method (Martella et al., 2017). All plasmids assembled using EMMA have a backbone consisting of an ampicillin resistance gene and a high-copy-number ColE1/pMB1/pBR322/pUC origin of replication. Backbone elements are denoted by terminating dotted lines in all plasmid schematics (Figures 1, 2 and Supplementary Figures 1–4). The EMMA toolkit was a gift from Yizhi Cai (Addgene kit # 1000000119) (Martella et al., 2017). The *lacI* CDS and C-terminal NLS were cloned from the Addgene plasmid pKG215 and inserted into an EMMA entry vector to create an EMMA part. pKG215 was a gift from Iain Cheeseman (Addgene plasmid # 45110) (Gascoigne et al., 2011). A frameshift mutation was introduced by inserting an adenosine in the fourth codon of *lacI* to create a non-functional LacI (NFLacI) for use in control experiments. The LacI-expressing module contains a minimal SV40 promoter, the *lacI* gene, and a SV40 polyA tail, with or without the addition of an upstream CMV enhancer (Figure 2). The repressible reporter plasmid includes a CMV enhancer and a minimal SV40 promoter upstream of a firefly luciferase gene with symmetric *lac* operators inserted in its 5′UTR, terminated by a SV40 polyA tail. To maximize repression activity, six copies of the *lac* operators containing the sequence AATTGTGAGCGCTCACAATT were utilized in this study. This sequence is the “symmetric” *lac* operator that possesses tighter binding with LacI than the canonical *lac* operator sequences (Lee et al., 2017).

### Cell Culture

The zebrafish fibroblast cell line PAC2 was maintained as previously reported (Senghaas and Köster, 2009). Cells were grown at 28°C in Leibovitz’s L–15 + glutamine Medium (Invitrogen, 21083027) containing 15% heat inactivated fetal bovine serum (FBS; Sigma-Aldrich, F4135–500 mL) and 1% antibiotic-antimycotic (Corning, MT30004CI) until confluent. Confluent cells were washed with 1x phosphate buffered saline (PBS; Invitrogen, 10010023) and detached from the plate with 0.05% Trypsin-EDTA for 5 min (Invitrogen, 25300054). Trypsin was quenched with FBS supplemented Leibovitz’s L–15 Medium and detached cells were distributed into sterile flasks with fresh media. PAC2 cells were obtained from the Antonellis Lab at the University of Michigan (RRID:CVCL_5853).

### Electroporation and Luciferase Reporter Assay

To assess the activity of the CMV enhancer in zebrafish cell culture, 4,000 ng of firefly luciferase expressing plasmids, either with or without the CMV enhancer, were transfected into 2 × 10^6^ PAC2 cells via electroporation (Figure 1). 100 ng of the renilla luciferase expressing plasmid (pRL-SV40 Promega, E2231) was included as a transfection control. Firefly/renilla luciferase signal was calculated as the mean of ratios of three technical replicates per biological replicate. Fold change was calculated relative to the signal of the SV40 promoter-only containing plasmid. The mean of fold-changes is reported and error bars represent standard deviation.

To test the functionality of our dual module *lac* repressor system in zebrafish cell culture, 2,000 ng of repressible module and 2,000 ng of LacI-expressing plasmid were co-transfected into 2 × 10^6^ PAC2 cells by electroporation. Four hundred nanogram of pRL-SV40 was included as a transfection control (Figure 2).

All transfections were completed using 2 mm cuvettes (Bulldog Bio, 12358–346) and electroporated using a NEPA21 Electroporator (Nepagene). Cells were harvested from culture and resuspended in 90 μL of Opti-MEM Reduced Serum Medium (Thermo Fisher Scientific, 31985062) per 1 × 10^6^ cells. Mastermixes of cells and DNA were prepared according to scale of conditions, and distributed into cuvettes (100 μL/cuvette, 10 μL of DNA, and 90 μL of cells). Poring pulse for PAC2 cells was set to the following: 200 V, Length 5 ms, Interval 50 ms, Number of pulses 2, D rate% 10, and Polarity +. Poring pulse for K562 was set to the following: 275 V, Length 5 ms, Interval 50 ms, Number of pulses 1, D rate 10%, and Polarity +. For both cell types, the transfer pulse conditions were set to the following: 20 V, Pulse length 50 ms, Pulse interval 50 ms, number of pulses 5, D rate 40%, and Polarity ±. Immediately following electroporation, each cuvette was recovered in 900 μL of appropriate media and distributed into a well on a 24-well culture plate. For the transfection in Figure 2, PAC2 cells were recovered in 6-well plates. Each condition had a total of 3 biological replicates. For experiments including LacI, IPTG was treated as a separate condition and added to 1 mm final concentration, unless otherwise specified, at 1 and 24 h post-transfection (Supplementary Figure 4).

Luciferase results were collected 48 h post-transfection on a GloMax-Multi + Detection System (Promega, E7081) using the Promega Dual-Glo Luciferase Assay System (Promega, E2940).

## Data Availability Statement

The original contributions presented in the study are included in the article/Supplementary Material, further inquiries can be directed to the corresponding author/s.

## Ethics Statement

This research was approved by the University of Michigan’s University Committee on Use and Care of Animals (protocol no. PRO00008385).

## Author Contributions

SN performed and analyzed the CMV validation in cells and whole fish. TM performed and analyzed the *lac* operator-repressor system validations in cells. GF and MH assisted in the *Iac* operator-repressor system experiments. MD and JS generated key components necessary to the completion of these experiments and substantially contributed to the design of this work. AB made substantial contributions toward the conception of this work. SN, TM, GF, MD, JS, and AB wrote the manuscript. All authors read and approved the final manuscript.

## Conflict of Interest

The authors declare that the research was conducted in the absence of any commercial or financial relationships that could be construed as a potential conflict of interest.
