# Supplementary material for: The Inducible lac Operator-Repressor System Is Functional in Zebrafish Cells
Source: Front Genet. 2021 Jun 18;12:683394. doi: 10.3389/fgene.2021.683394 (PMC8249864; doi:10.3389/fgene.2021.683394)
Supplement: Supplementary file 1 [file Presentation_1.pdf]

## Supplementary Materials

# The inducible *lac* operator-repressor system is functional in zebrafish cells

**Sierra S. Nishizaki[1,2,*], Torrin L. McDonald[1,*], Gregory A. Farnum[2], Monica J. Holmes[2], Melissa L. Drexel[1], Jessica A. Switzenberg[2], Alan P. Boyle[1,2,†]**

**1. Department of Human Genetics, University of Michigan, Ann Arbor, MI, USA 48109**

**2. Department of Computational Medicine and Bioinformatics, University of Michigan, Ann Arbor, MI, USA 48109**

*** These authors contributed equally to this work.**

**† Correspondence: apboyle@umich.edu**

## Supplemental Methods

### Zebrafish microinjections

Tol2 enhancer assay plasmids for whole zebrafish injection with CMV were assembled using the universal expression plasmid (pGW_*cfos*eGFP) and methodologies published from Fisher *et al*. 2006 (Fisher et al., 2006).

Microinjections were carried out using the *Tol2* transposon system as previously described (Kawakami, 2007; Suster et al., 2009). Zebrafish embryos were co-injected within 30 minutes of fertilization in the single-cell stage using a mix of 1uL 25ng/uL *Tol2* mRNA, 1uL 125ng/uL experimental plasmid, 0.5uL phenol red for visualization and 2.5uL DNase/RNase free water. All embryos were maintained in 1X Holtfreter's buffer (Holtfreter Buffer, 2013) and fluorescent activity was assessed at 24 and 48 hours post-fertilization.

### Cell Culture

The human myelogenous leukemia cell line K562 was cultured in RPMI 1640 + glutamine (ThermoFisher, 11875093) supplemented with 10% heat inactivated fetal bovine serum and 1% antibiotic-antimycotic until confluent. During growth K562 cells were incubated at 37°C in 5% $CO_2$. Cells were split every two days into fresh media to avoid extended confluence.

### Electroporation and Luciferase Reporter Assay

To determine the optimal amount of IPTG, we co-transfected 400ng of repressible reporter plasmid and 400ng of LacI-expressing plasmid into $1x10^6$ K562 cells by electroporation. Cells were recovered in supplemented media containing 0, 1, 2.5, 5, 10, 20, or 50mM of IPTG. 75ng of a Renilla luciferase expressing plasmid (pRL-SV40) was included as a transfection control (**Sup. Fig. 4**).

To compare the LacI dual module between different human and zebrafish cell types, 500ng of the repressible module was co-transfected with 250ng or 500ng of LacI-expressing plasmids into $1x10^6$ K562 cells or PAC2 cells by electroporation. 100ng of pRL-SV40 plasmid was included as a transfection control (**Sup. Fig. 2, Sup. Fig. 3**).

**Supplemental Figures**

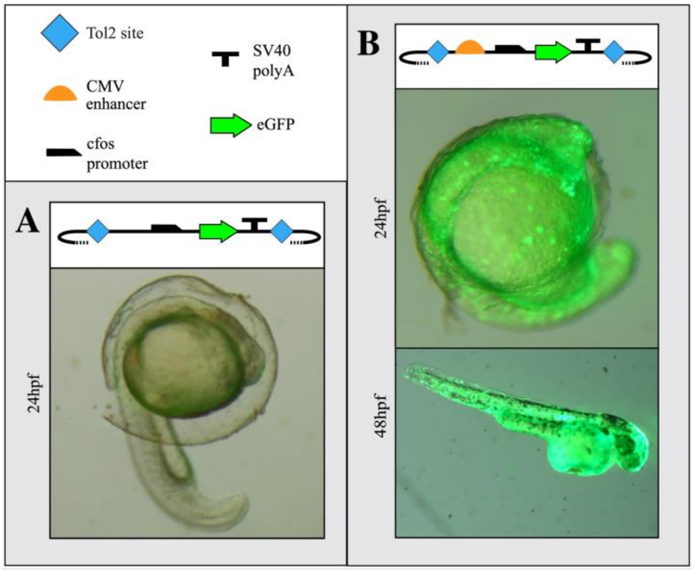


**Supplemental Figure 1.** The CMV enhancer drives strong widespread reporter gene expression in zebrafish embryos. A) SV40 promoter-only plasmids did not result in observable eGFP expression in 24hours post-fertilization zebrafish embryos (0/76 GFP+). B) CMV enhancer driving eGFP shows widespread expression in zebrafish up to 48 hours post-fertilization (63/69 GFP+ at 24hrs, 49/49 GFP+ at 48hrs). All plasmid components for each transfection design are detailed as symbols at the top of the figure. The TSS starts where the cfos promoter begins to slope downward.

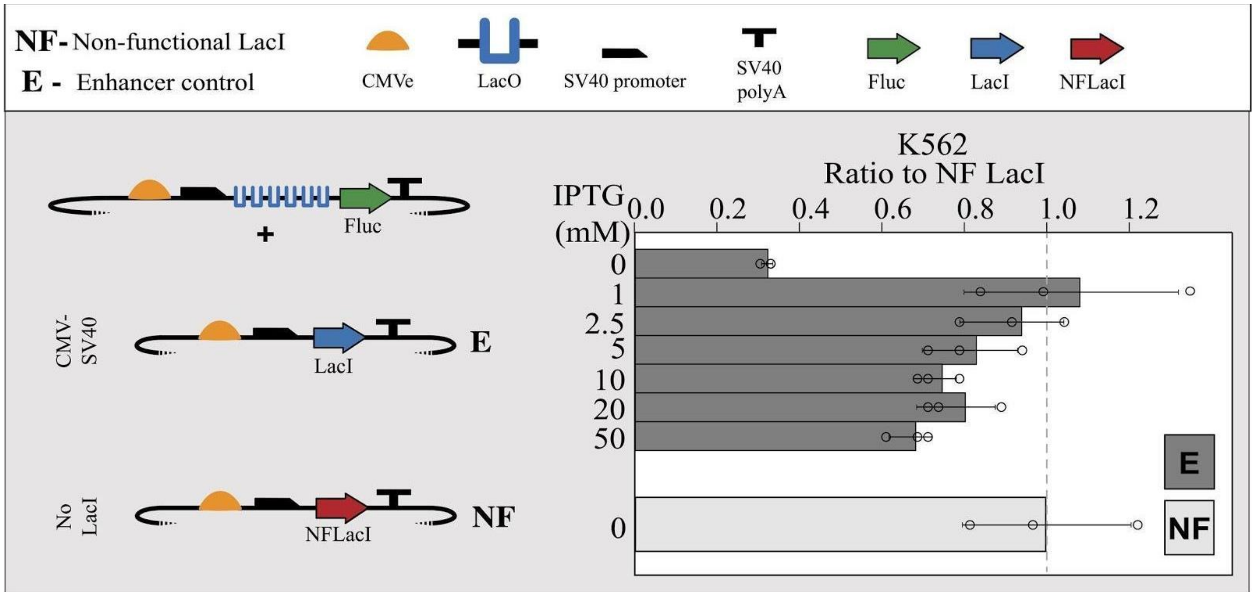


**Supplemental Figure 2.** High levels of IPTG negatively impact the output of the *lac* operator-repressor system in K562 cells. Co-transfections of repressible reporter plasmids with equimolar amounts of CMV enhancer enhancer driven LacI-expressing plasmids were exposed to increasing levels of IPTG. At 1mM IPTG, the output signal is restored to the level observed when NFLacI-expressing plasmids are co-transfected with repressible reporter plasmids, indicating that 1mM IPTG is sufficient to relieve LacI repression. At IPTG concentrations >1mM an increasingly lower output of signal relative to NFLacI levels was observed. The TSS starts where the SV40 promoter begins to slope downward. Error bars represent standard deviation of replicates (n=3). Points represent values for all 3 replicates in each condition. The dashed line shows the NF LacI IPTG- negative control.

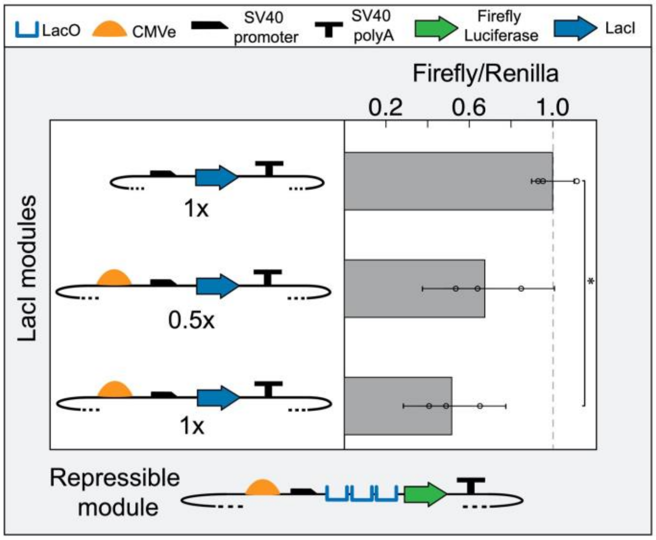


**Supplemental Figure 3.** Increasing concentrations of LacI positively correlates with increased luciferase repression. By co-transfecting half of the concentration of LacI in K562 cells we found contributed ~30% less repression of luciferase than cells transfected with equal concentrations of LacI (~67% for 0.5x, ~51% for 1x transfections). All plasmid components for each transfection design are detailed as symbols at the top of the figure. The TSS starts where the SV40 promoter begins to slope downward. Error bars represent standard deviation of replicates (n=3). Points represent values for all 3 replicates in each condition. The dashed line shows expression level of the promoter-only negative control. Statistical significance was determined using a Student's two tailed t-test * P-score < 0.01. No statistically significant difference was noted between the promoter-only control and the 0.5X LacI transfection.

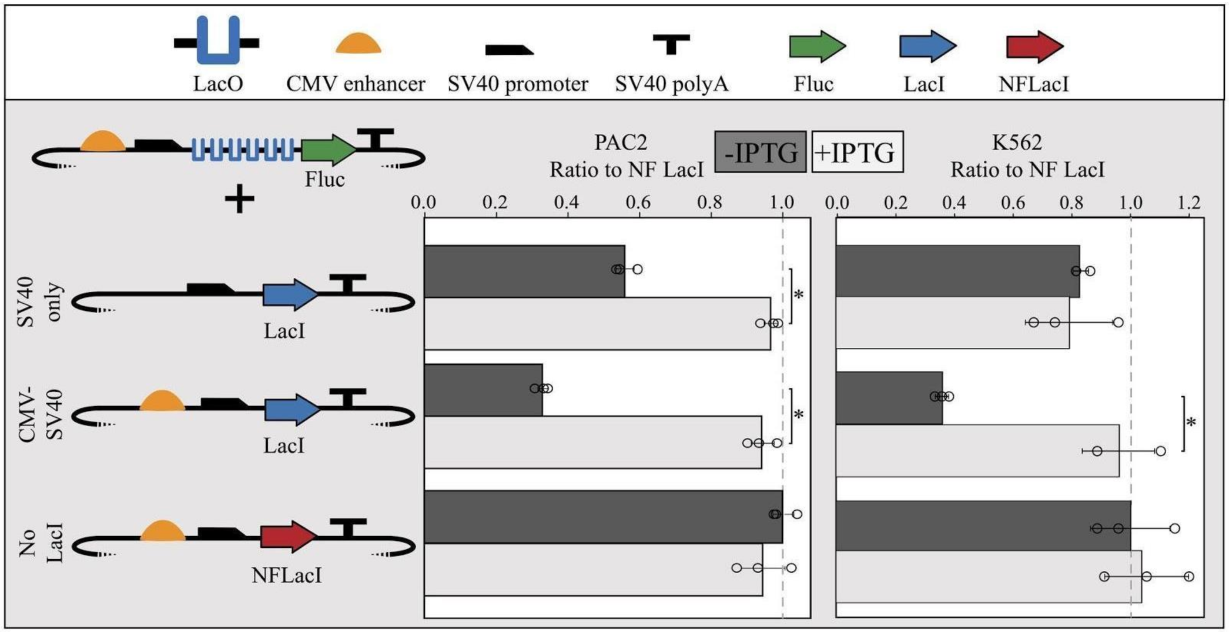


**Supplemental Figure 4.** The *lac* operator-repressor system performs similarly in both human and zebrafish cell lines. K562 and PAC2 cells transfected with the same *lac* operator-repressor plasmid mixtures result in similar repression profiles. The promoter-only LacI-expressing plasmids resulted in roughly 10-20% repression in both cell types and the CMV-SV40 enhancer-promoter driven LacI-expressing plasmids resulted in ~60% repression in both cell types. For each of the 3 plasmid combinations above, 100ng of pRL, 4:1 molar equivalents of repressible module plasmid:pRL, and 4:1 molar equivalents of LacI-expressing plasmid:pRL were co-transfected into 1 million K562 cells or PAC2 cells. 6 biological replicates were performed for each condition and 3 were exposed to 1mM IPTG and the remaining 3 were not exposed to IPTG. The TSS starts where the SV40 promoter begins to slope downward. Error bars represent standard deviation of replicates (n=3). Points represent values for all 3 replicates in each condition. The dashed line shows the NF LacI IPTG- negative control. Statistical significance was determined using a Student’s two tailed t-test * P-score < 0.001.
